# Tomato Infection by Whitefly-Transmitted Circulative and Non-Circulative Viruses Induce Contrasting Changes in Plant Volatiles and Vector Behaviour

**DOI:** 10.3390/v8080225

**Published:** 2016-08-11

**Authors:** Alberto Fereres, Maria Fernanda G. V. Peñaflor, Carla F. Favaro, Kamila E. X. Azevedo, Carolina H. Landi, Nathalie K. P. Maluta, José Mauricio S. Bento, Joao R.S. Lopes

**Affiliations:** 1 Departamento de Protección Vegetal, ICA-CSIC, c/Serrano 115 dpdo., Madrid 28006, Spain; 2 Department of Entomology, Federal University of Lavras, Lavras, MG 37200-000, Brazil; fernanda.penaflor@gmail.com; 3 Departamento de Ciências Exatas e Tecnologicas, Universidade Estadual de Santa Cruz, Ilheus, BA 45662-900, Brazil; carlaffavaro@gmail.com; 4 Department of Entomology and Acarology, University of Sao Paulo, ESALQ-USP, Piracicaba, SP 13418-900, Brazil; kamilaazevedo30@hotmail.com (K.E.X.A.); carolhlandi@gmail.com (C.H.L.); nathalie_maluta@yahoo.com.br (N.K.P.M.); jmsbento@usp.br (J.M.S.B.); jrslopes@usp.br (J.R.S.L.)

**Keywords:** whitefly vector, *Tomato chlorosis virus*, *Tomato severe rugose virus*, plant volatiles, vector-borne plant viruses, host plant selection

## Abstract

Virus infection frequently modifies plant phenotypes, leading to changes in behaviour and performance of their insect vectors in a way that transmission is enhanced, although this may not always be the case. Here, we investigated *Bemisia tabaci* response to tomato plants infected by *Tomato chlorosis virus* (ToCV), a non-circulative-transmitted crinivirus, and *Tomato severe rugose virus* (ToSRV), a circulative-transmitted begomovirus. Moreover, we examined the role of visual and olfactory cues in host plant selection by both viruliferous and non-viruliferous *B. tabaci*. Visual cues alone were assessed as targets for whitefly landing by placing leaves underneath a Plexiglas plate. A dual-choice arena was used to assess whitefly response to virus-infected and mock-inoculated tomato leaves under light and dark conditions. Thereafter, we tested the whitefly response to volatiles using an active air-flow Y-tube olfactometer, and chemically characterized the blends using gas chromatography coupled to mass spectrometry. Visual stimuli tests showed that whiteflies, irrespective of their infectious status, always preferred to land on virus-infected rather than on mock-inoculated leaves. Furthermore, whiteflies had no preference for either virus-infected or mock-inoculated leaves under dark conditions, but preferred virus-infected leaves in the presence of light. ToSRV-infection promoted a sharp decline in the concentration of some tomato volatiles, while an increase in the emission of some terpenes after ToCV infection was found. ToSRV-viruliferous whiteflies preferred volatiles emitted from mock-inoculated plants, a conducive behaviour to enhance virus spread, while volatiles from ToCV-infected plants were avoided by non-viruliferous whiteflies, a behaviour that is likely detrimental to the secondary spread of the virus. In conclusion, the circulative persistent begomovirus, ToSRV, seems to have evolved together with its vector *B. tabaci* to optimise its own spread. However, this type of virus-induced manipulation of vector behaviour was not observed for the semi persistent crinivirus, ToCV, which is not specifically transmitted by *B. tabaci* and has a much less intimate virus-vector relationship.

## 1. Introduction

An increasing number of studies demonstrate that vector-borne parasites are able to manipulate several phenotypic traits of their vertebrate or plant hosts and vectors in ways that render parasite transmission more likely (reviewed by [1,2,3]). Pathogen-induced changes in plant phenotype often result in increased attraction and altered performance of vectors on infected plants, which can result in a mutualistic, antagonistic, or neutral type of interaction (reviewed by [4,5]). Attraction of infected host plants to arthropod vectors is not a phenomenon restricted to plant viruses. For vertebrate pathogens, increased attraction and arrestment of mosquitoes and ticks to diseased hosts have also been observed [1].

The existing literature on the effects of plant virus infection on insect behaviour has mainly concentrated on aphid-transmitted viruses. Most of the studied vector-borne plant viruses have been shown to modify vector behaviour primarily indirectly, by promoting physiological and morphological changes in virus-infected plants that increase plant attractiveness and preference to aphid vectors [5,6,7]. Thus, viral infection often causes visual symptoms (e.g., yellowing leaves), which are known to attract insect vectors, especially aphids [8,9]. However, more recently it has been found that long-range olfactory cues, such as volatile organic compounds (VOCs) emitted from virus-infected plants, differ from healthy plants, and are important mediators in the attraction and decision-making of insect vectors when selecting a host plant. For example, aphids were attracted to volatiles produced by plants infected with the non-persistently-transmitted *Cucumber mosaic virus* (CMV, *Bromoviridae*), but once aphids landed on and probed the infected plants, they quickly left the plant and avoided prolonged feeding, a conducive behaviour that enhances the spread of non-persistent viruses [7]. Conversely, persistently-transmitted viruses often promote long term feeding as shown for the aphid vector *Sitobion avenae* (Hemiptera: Aphididae), which engaged in faster phloem-finding behaviour and longer phloem feeding periods on plants infected with *Barley yellow dwarf virus* (BYDV, *Luteoviridae*) than on non-infected plants [10,11]. More recent studies show that plant viruses can directly alter the aphid host selection, by reversing their preference for BYDV-infected plants after virus acquisition during in vitro feeding [12]. Similarly, non-viruliferous aphids preferred to settle on *Potato leaf roll virus* (PLRV, *Luteoviridae*)-infected plants, while viruliferous aphids preferred sham-inoculated plants, a behaviour that at least at the initial stages is mediated by plant volatile emissions [13]. However, recent studies show that not all virus-vector-plant interactions are conducive to transmission. For instance, soybean plants infected with the potyvirus *Soybean mosaic virus*, SMV, reduced the population growth of *Aphis glycines* but increased aphid preference for infected plants, a behaviour that is not conducive to the non-persistent transmission of SMV [14].

Besides being attractants of vectors, modification of the VOC blend may influence arresting behaviour, an effect that is important for the acquisition of persistently-transmitted viruses. For example, the aphid *Myzus persicae* was more attracted to potato leaves infected by PLRV than to non-infected leaves because the infected-plant volatile blend acted as a stronger attractant and arrestant than those emitted bynon-infected plants [6]. Studies on the interactions between BYDV-infected plants and the aphid vector *Rhopalosiphum padi* suggests that attraction rather than arrestment by VOCs is the underlying mechanism in the preference of non-viruliferous aphids for virus-infected plants [15]. It is also known that nutritional composition of virus-infected plants may favour aphid development and reproduction. An increased concentration of free amino acids in the sap of BYDV-infected plants [16] were likely involved in the higher performance of *S. avenae* on BYDV-infected plants [17].

In contrast to the understanding of aphid-borne plant viruses, little is known about the role of VOCs and visual cues in whitefly-virus-plant interactions. There is evidence that olfactory cues can play a role on the host plant finding behaviour of whiteflies. For example, the whitefly *Bemisia tabaci* (Gennadius) (Hemiptera: Aleyrodidae) perceives tomato constitutive volatiles as a positive stimulus, indicating that plant odours constitute guiding cues for host plant selection. The same work showed that coriander VOCs reduced the attractiveness of tomato volatiles, but was not a repellent for *B. tabaci* [18]. Furthermore, Zhang et al. found that ginger oil can act as a strong repellent to *B. argentifolii* [19] (currently known as theMiddle East-Asia Minor one, MEAM1, species of *B. tabaci*).

In addition to volatiles, it is well known that whiteflies use visual cues during the host plant finding process. Mound described two ranges of the spectrum, yellow and blue/ultraviolet, to which *B. tabaci* is particularly sensitive [20]. Yellow is particularly attractive to whiteflies, and this is the reason why yellow sticky traps have been used for many years to monitor the population levels and dispersion patterns of *B. tabaci* [21]. Furthermore, studies conducted by Antignus et al. showed that *B. tabaci* is attracted by 254–366 nm radiation when exposed to monochromatic UV sources as well as to full-spectrum light [22]. In fact, UV-blocking materials are known to reduce take-off, orientation, and navigation ability of *B. tabaci*.

*Bemisia tabaci* is a vector of more than 110 plant viruses as well as a serious pest of many horticultural crops worldwide [23,24,25]. This insect inflicts damage both directly, by feeding on the phloem, and indirectly by transmitting a wide array of viruses, most notably begomoviruses transmitted in a persistent-circulative manner and criniviruses, transmitted in a semi-persistent-non-circulative manner.

While begomoviruses circulate through the vector’s haemolymph and salivary glands, criniviruses do not have such an intimate relationship with its vector, and such differences in the level of “intimacy” may influence vector behaviour in different ways. To test this hypothesis, we selected *Tomato severe rugose virus* (ToSRV, *Geminiviridae*), the predominant begomovirus infecting tomatoes in Brazil [26], and *Tomato chlorosis virus* (ToCV, *Closteroviridae*) a worldwide distributed crinivirus causing serious disease in tomatoes and other solanaceous crops [25]. The aim of our work was to investigate if ToSRV and ToCV manipulate the behaviour of its vector, *B. tabaci*, in ways that can affect virus spread. Furthermore, we examined whether visual and/or olfactory cues of tomato plants infected with ToSRV and ToCV play a role on the host plant selection behaviour of both viruliferous and non-viruliferous *B. tabaci* adults. In addition, we chemically characterised the composition of volatile compounds emitted by virus-infected and mock-inoculated plants. Such knowledge about volatiles involved in attraction/settlement can be relevant for the development of defence strategies against *B. tabaci* (e.g., by silencing genes involved in the synthesis of attractive plant volatiles).

## 2. Material and Methods

### 2.1. Plants, Insects, and Virus Isolates

We used tomato plants (*Lycopersicon esculentum* Mill. cv. Santa Clara) grown in 1.5-Lplastic pots with Tropstrato HT (Vida Verde Ltda., Mogi Mirim, SP, Brazil) potting mix, watered daily and fertirrigated weekly with a suspension of macro (N-P-K: 10-30-10) and micronutrientes (1.5 g/L). Plants and insect colonies were produced in greenhouse facilities (26 ± 4 °C and 70 ± 20 relative humidity (RH)) at Luiz de Queiroz College of Agriculture (ESALQ), University of São Paulo, in Piracicaba, SP, Brazil.

A virus-free colony of *B. tabaci* MEAM1 species (former B biotype), originated from Instituto Agronômico de Campinas (IAC, Campinas, SP, Brazil) in 2013, was maintained on cabbage (*Brassica oleracea*L. var. *acephala* DC. cv. Manteiga) in cages covered by insect-proof netting. After several generations on cabbage, whiteflies were acclimated to tomato plants for one generation before experiments began. To obtain viruliferous whiteflies for the experiments, 1-week old adults from the rearing colony were enclosed in clip-cages on ToCV or ToSRV-infected tomato plants for a 72-h acquisition access period (AAP). Similarly, whiteflies used as non-viruliferous controls were enclosed in clip-cages attached to virus-free plants for the same 72-h period.

The ToSRV and ToCV isolates were obtained from tomato plants originally collected in Piracicaba and Sumaré, SP, Brazil, respectively. To obtain virus-infected tomato plants, 20–25 non-viruliferous adult whiteflies were confined in clip-cages on virus-infected tomato plants (ToSRV or ToCV) for a 72-h AAP, and then transferred to healthy (two true-leaf) tomato plants for a 5-day inoculation access period (IAP). The infested leaf was excised from the plant together with the clip cage to eliminate any eggs laid by adults during the IAP. The same procedure was used for obtaining healthy (mock-inoculated) tomato plants, except that non-viruliferous whiteflies were placed on non-infected plants. Virus-inoculated plants were checked by PCR 3 weeks after inoculation to verify infection status. Similarly, infection status of viruliferous whiteflies was also regularly checked by PCR. Nucleic acid extractions and PCR were conducted as described previously for ToSRV [27,28] and ToCV [29]. For ToCV, Total RNA extraction was done with Trizol^®^ LS (Invitrogen, Carlsbad, CA, USA), according to the manufacturer information. Only infected plants that developed clear diagnostic symptoms of the viruses were used in the experiments. Virus-infected and mock-inoculated plants were grown under the same environmental conditions, but in separate compartments (isolated by plastic walls and vector-proof screens) of a greenhouse to avoid contamination, and used in experiments four weeks after virus inoculation.

### 2.2. Free-Choice Test to Assess Visual Stimuli of Virus-Infected and Mock-Inoculated Plants during Whitefly Landing

Free-choice assays were conducted to assess whitefly response during landing to visual cues coming from either virus-infected or mock-inoculated plants. The procedure was similar to that previously described in [30]. A flight release chamber and a landing platform was constructed and placed inside a cage made with insect-proof netting (75 cm × 75 cm × 115 cm) (Bugdorm, BD2400F, MegaView Science Co., Ltd., Taichung Taiwan). A brown cardboard surface (66 × 66 cm) was divided into 36 squares (11 × 11 cm) arranged in four replicates of the same 3 × 3 Latin square design. The design was configured in a way that avoided surrounding any given target by another of the same type. Thus, leaves of virus-infected plants with characteristic symptoms and from mock-inoculated plants together with blanks (brown background resembling the soil colour) were used as landing targets (Figure S1). Then, a Plexiglas sheet coated with mineral oil was placed on top of the leaves to provide a uniform landing surface and avoid any leaf olfactory cues. Groups of 50 adult whiteflies from the colony were transferred to each of the five glass tubes of a flight release platform (a total of 250 whiteflies, Figure S2). Then, the platform was quickly suspended with strings from the centre of the cage at a height of 1 m before the whiteflies started to fly. The cages and landing platform with the specific targets were mounted in a greenhouse (28 ± 4 °C, 70% ± 20% RH) just before experiments began. Whiteflies were released from the flight platform at 11:00 a.m. by gently removing the lid of each glass vial. The number of whiteflies landing on the targets was counted 6 h after release. Experiments were conducted using a platform covered by either ToSRV or ToCV-infected leaves versus mock-inoculated leaves and the blanks. Each experiment was replicated four times using either viruliferous or non-viruliferous whiteflies.

The number of whiteflies landing on each target were transformed by ln(x+1) before analysis to reduce heteroscedasticity and were then subjected to an analysis of variance (ANOVA) using Statview software [31] to determine if there were any significant (*p* < 0.05) differences between treatments. Means were separated by the Fisher protected least significant difference (LSD) test.

### 2.3. Whitefly Preference for Virus-Infected and Mock-Inoculated Plants in the Presence and Absence of Light

A series of dual-choice tests under illuminated and dark conditions were designed to assess if whiteflies responded to headspace volatiles emitted by virus-infected plants. The test arena was made of a 12 cm-diameter Petri dish with its base divided in two halves. Each section of the base had a 2 × 5 cm rectangular hole covered with a whitefly-proof nylon screen. The abaxial surface of a non-excised leaf of either a virus-infected or mock-inoculated plant was positioned on the top of the rectangular holes of the Petri dish, a method adapted from Eigenbrode et al. [6]. Another set of assays was conducted, testing whitefly preference to mock-inoculated leaves on one side and blank (no leaf) on the other. All tests were conducted under a cool white light-emitting diode (LED) light or an infrared light source to provide either illuminated or dark conditions, respectively (Figure S3). The infrared light was used to track whitefly movement under dark conditions without disturbing insect behaviour. Twenty adult whiteflies were enclosed in a glass vial placed in an ice bath for 5 min to facilitate manipulation. Whiteflies were then released in the middle of the Petri dish, the lid was closed and the abaxial surface of the last expanded tomato leaf was placed facing the rectangular hole of the Petri dish (Figure S3). The number of times that each whitefly entered the selected test surface covered by the nylon screen was recorded during a 10 min period. We used a mirror below the Petri dish lid to facilitate observation of whitefly movement as they were walking upside down across the test arena. Our experimental set-up provided a way for whiteflies to respond to visual cues (in the presence of light) and/or to headspace volatiles (in the absence of light) from either virus-infected or mock-inoculated leaves. Each assay was repeated 20 times using five sets of 20 whiteflies/test and four different test plants per treatment under illuminated and dark conditions. All these tests were conducted with non-viruliferous whiteflies.

Numbers of whiteflies responding to the treatments under light and dark conditions were analysed by a chi-square test (*p* < 0.05) using a general log-linear model (GLM) and following a quasi-poisson distribution. The analysis was performed using software package R 3.1.0 [32].

### 2.4. Active Airflow Olfactometer Tests to Assess Whitefly Preference to VOCs Emitted by Virus-Infected and Mock-Inoculated Tomato Plants

A series of preliminary Y-tube olfactometer designs were tested to find out if VOCs emitted by tomato plants were perceived by *B. tabaci*. Different conditions and variables were tested including olfactometer orientation, air-flow pressure, number of whiteflies, whitefly sex, olfactometer diameter, temperature, and illumination. After these preliminary tests we were able to establish an experimental set-up that provided reliable whitefly response to volatiles emitted by mock-inoculated tomato plants (a significantly higher number of whiteflies responding to tomato plants when compared to the blank).

The final set-up used to assess whitefly response to volatiles was based on a glass Y-tube olfactometer (main arm: 9 cm long and diameter of 4 cm; side arms: 10 cm long, diameter of 4 cm) horizontally positioned. Each lateral arm was connected to a hermetically-sealed glass chamber (38 × 21 cm) containing the test plants and then to an ARS^®^ volatile collection system (Analytical Research Systems, Gainesville, FL, USA), which pushed air through the chambers and olfactometer. Air flow was adjusted using flowmeters to 0.2 L/min/arm. Flexible tubes made of polytetrafluorethylene (PTFE) were used for all connections of the olfactometer system. External walls of the plant chambers were covered with filter paper to avoid whitefly detection of visual cues from the tomato plants. The Y-tube olfactometer was rotated 180° after each replicate to avoid positional bias and supplementary lights were placed above the plant chambers to provide uniform light during the testing period (Figure S4). All tests were conducted in laboratory conditions at 25 ± 2 °C and RH of 70% ± 10%, between 10:00 a.m. and 5:00 p.m. Olfactometers were washed with acetone, rinsed with hexane and dried in the oven at 170 °C for 20 min. Plant chambers were washed using a neutral detergent and dried out with a paper towel.

Whiteflies, starved for 2 h, were individually released in the main olfactometer arm and their response was recorded for up to 15 min. We considered a choice as when whiteflies crossed a line established at 8 cm from each lateral arm and remained across the border line for more than 3 min. A black cardboard was placed below the olfactometer to facilitate the observation of whitefly movement.

Whitefly preference was assessed when exposed to odours emitted by: (i) mock-inoculated tomato plant vs. clean air (empty glass chamber); (ii) ToSRV-infected plant vs. mock-inoculated plant; (iii) ToCV-infected plant vs. mock-inoculated plant. Experiments were conducted using either viruliferous or non-viruliferous adult whiteflies. A total of 70 individual whiteflies (replicates) were tested for each assay. Whiteflies not choosing a side arm within 15 min were considered as non-responsive and were excluded from data analysis. Each whitefly was tested only once.

The choices of the non-viruliferous and viruliferous whiteflies in the olfactometer were analysed by means of a chi-square goodness of fit test for each of the two pairwise comparisons (mock-inoculated tomato vs. blank, ToSRV-infected vs. mock-inoculated tomato, and ToCV-infected vs. mock-inoculated tomato). A *p*-value of 0.05 was considered significant and tests were run using the Statview software [31].

### 2.5. Volatile Collection and Analysis from Virus-Infected and Mock-Inoculated Plants

ToSRV-infected, ToCV-infected and mock-inoculated tomato plants were enclosed in glass chambers connected to the ARS^®^ Volatile Collection System (Figure S5). Clean and humidified air was pushed through the system at 1.3 L/min/chamber and a vacuum pump pulled air at 1.0 L/min/chamber. Volatiles from three ToCV-infected and six ToSRV-infected plants, with their respective mock-inoculated tomatoes, were collected under supplementary light for a total of 15 h (from 9:00 a.m. to 4:30 p.m. for two consecutive days) and trapped in a column containing 30 mg of adsorbent polymer HayeSep-Q 80/100 Mesh (Alltech Assoc., Columbia, MD, USA). Polymer trap was then eluted with 180 µL of bi-distilled hexane containing dodecane at 5 ng/mL as an internal standard. An aliquot of 1 µL from each sample was analysed using gas chromatography coupled to mass spectrometry (GC-MS) (Varian 4000, Varian Inc., Palo Alto, CA, USA) equipped with a HP5-MS capillary column (30 m × 0.25 mm × 0.25 μm), and using helium as a carrier gas. The column temperature was held at 40 °C for 2 min, and gradually increased at a rate of 8 °C/min until reaching 200 °C. Then, the temperature was raised at a rate of 20 °C/min until it reached 250 °C where it was held for 2 min. Compounds were identified by comparing the obtained mass spectra retention times with those of the library (NIST 98) and authentic standards as well as the Kovats Index (KI) using *n*-alkane (C7- C30) standards.

Compound quantification was estimated based on the peak area relative to the internal standard. Plant volatile composition was analysed by MANOVA (*p* < 0.05), when possible, as well as by principal component analysis (PCA). The amounts of individual compounds that followed a Gaussian distribution were analysed by Student’s *t*-test, while those that followed a non-Gaussian distribution were analysed by Welch’s test (*p* < 0.05).

## 3. Results

### 3.1. Virus-Infected Leaves Are Visually More Attractive to Whiteflies during Landing

Most of the whiteflies landed on the Plexiglass plate 6 h after release. The total mean number of whiteflies recaptured was 205.75 ± 45.96 and 171.62 ± 50.79 for the ToCV and ToSRV assays, respectively. The landing rate was significantly higher on the ToSRV-infected and mock-inoculated leaves than on the blank (brown background) targets for both non-viruliferous and viruliferous whiteflies (Table 1). The blank (brown colour) was by far much less attractive than the tomato leaf targets. Twice as many non-viruliferous or viruliferous whiteflies preferred to land on ToSRV-infected than on mock-inoculated leaf targets (Table 1a). Similarly, non-viruliferous and viruliferous whiteflies landed preferentially on ToCV-infected leaves rather than on mock-inoculated leaves (Table 1b). Whiteflies landed at higher rates on both ToCV-infected and mock-inoculated leaves rather than on the blank (brown) targets, although no significant difference between mock-inoculated and the blank targets was observed for viruliferous whiteflies. Overall, our results show that whiteflies have a strong preference to land on virus-infected leaves in the absence of olfactory cues.

### 3.2. Visual Cues Are More Important Than Olfactory Stimuli to Whiteflies When Searching for Tomato Leaves

The dual-choice leaf arena test with no active air flow showed that whiteflies equally chose mock-inoculated leaf and control (blank, no leaf) in dark conditions; however, many more *B. tabaci* (7.4-fold) responded to mock-inoculated tomato leaves over the blank under illuminated conditions (Table 2). Whiteflies showed no preference for either virus-infected (ToSRV or ToCV) or mock-inoculated leaves under dark conditions, but a significantly higher number of whiteflies preferred ToSRV-infected leaves over mock-inoculated leaves in the presence of light. Furthermore, there was no evidence that whiteflies responded to any olfactory cues emitted from tomato plants because whiteflies showed no preference for either ToCV- or ToSRV-infected plants relative to mock-inoculated tomato leaves under dark conditions. In contrast, higher responsiveness of whiteflies was observed in the presence of light rather than in the dark (Table 2).

### 3.3. Whiteflies Are More Attracted to VOCs Emitted by Mock-Inoculated Than to Those of Virus-Infected Tomato Plants, but React in Different Ways Depending on Virus Type (Circulative or Noncirculative) and Whitefly Infection Status (Viruliferous or Non-Viruliferous)

**Figure 1:**
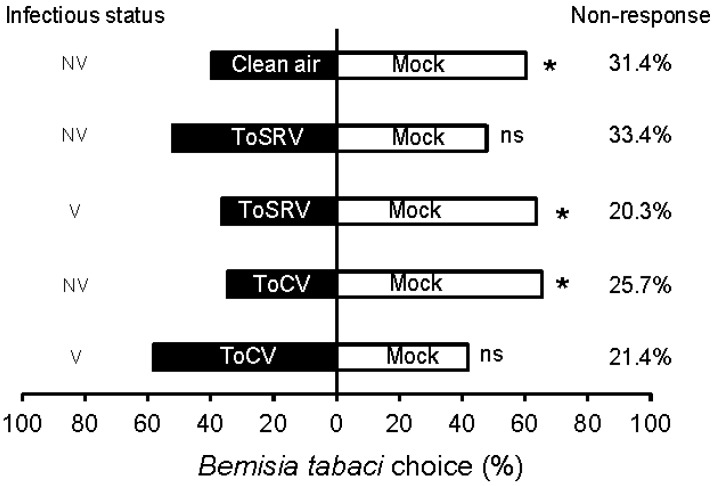
Percentage of whiteflies (%) responding to volatiles coming from different plant sources connected to a Y-tube olfactometer with a continuous active air flow. Individual adult whiteflies were released in the principal arm and their response was recorded in a 15 min time interval. A positive response was considered when whiteflies remained for 3 min across the border line of the lateral tubes. A total number of 70 replicates (whiteflies) per test were used. V: viruliferous whiteflies; NV: non-viruliferous; ns: not significant; *: *p* < 0.05.

Whiteflies preferred volatiles emitted from mock-inoculated tomato plants over clean air (blank) (χ^2^ = 4.17; *p* = 0.04), suggesting that whiteflies are able to respond to volatiles coming from their host plant when a continuous air flow was used (Figure 1).

Whiteflies responded differently to volatiles emitted by virus-infected plants depending on the virus type (ToSRV or ToCV) and on their infectious status (viruliferous or non-viruliferous). Non-viruliferous whiteflies showed no preference when exposed to odours from ToSRV-infected plants and mock-inoculated plants (χ^2^ = 0.17; *p* = 0.677). In contrast, viruliferous whiteflies oriented preferentially to odour cues from mock-inoculated over ToSRV-infected plants (χ^2^ = 8.18; *p* = 0.004).

Interestingly, the response of non-viruliferous and viruliferous whiteflies to volatiles emitted by ToCV-infected plants was totally different than the pattern observed for ToSRV-infected plants. Non-viruliferous *B. tabaci* preferred volatiles from mock-inoculated plants (χ^2^ = 9.85; *p* = 0.002), while viruliferous whiteflies showed no preference (χ^2^ = 2.94; *p* = 0.086).

### 3.4. Specific Terpenes Are Partially Suppressed after ToSRV-Infection but Others Augmented after ToCV-Infection

**Figure 2:**
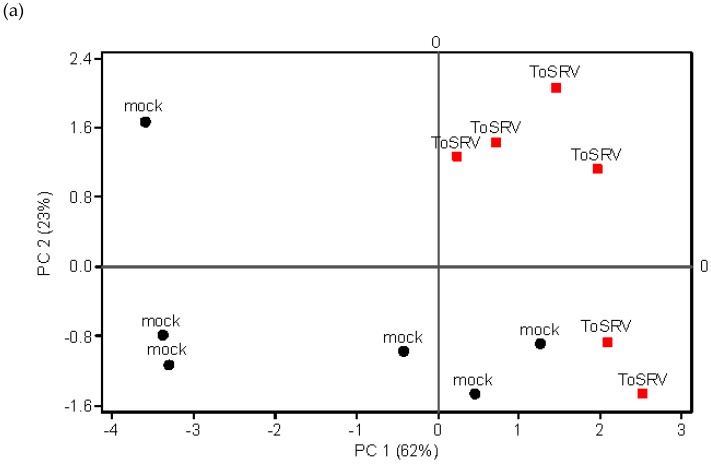

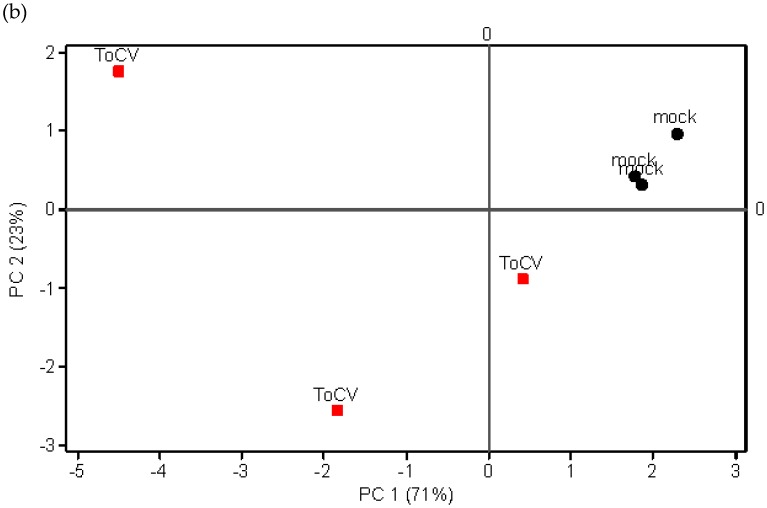
Loading plots for principal component analysis (PCA) with volatile compounds emitted by mock-inoculated and virus-infected plants. (**a**) mock-inoculated and ToSRV volatile emissions; (**b**) mock-inoculated and ToCV volatile emissions.

GC-MS analysis revealed only quantitative differences between volatile profiles emitted from virus-infected (ToSRV or ToCV) and mock-inoculated plants. Volatile composition emitted by ToSRV-infected plants differed from mock-inoculated plants (MANOVA, Wilk’s criterion, *F*_1,10_ = 96.5 *p* = 0.002), although PCA did not show clear separation between treatments (Figure 2) along principal component 1 (PC1) and principal component 2 (PC2). Analysis of individual compounds showed that mock-inoculated tomato plants emitted significantly higher amounts of the terpenes α-pinene, 4-carene, α-phellandrene, terpinene, and β-phellandrene than ToSRV-infected plants (Table 3a, Welch’s test, *p* < 0.05), indicating that ToSRV infection promotes suppression of some volatile terpenes.

Volatile collection from ToCV-infected plants revealed a wider range of compounds compared to the blend composition of the previous experiment with ToSRV (Table 3b). The number of replicates of ToCV-infected plants was not compatible with the number of compounds in the blend for running MANOVA, but PCA partially separated the volatile composition blend emitted by ToCV-infected from mock-inoculated plants (Figure 2). Unlike ToSRV infection, analysis of individual compounds showed that the volatile blend emitted by ToCV-infected plants comprises higher amounts of the terpenes β-caryophyllene, α-humulene, and two unidentified terpenes relative to mock-inoculated plants (Table 3b, Student’s test, *p* < 0.05).

## 4. Discussion

Plant viruses have co-evolved with their insect vectors inducing specific direct or plant-mediated changes in their behaviour or life history, leading in some cases to enhanced virus transmission and benefits in vector fitness [7,12,13,33,34,35]. This is well documented in a series of studies, however the mechanisms that drive such interactions are largely unknown [2].

Our study shows that when *B. tabaci* adults approach virus-infected plants, visual cues play a key role on their choice during landing. Our free-choice experiments show that *B. tabaci* preferred to land two-folds more often on ToSRV-infected than on mock inoculated leaves in the absence of olfactory cues (Table 1). Similar results were obtained with ToCV-infected leaves suggesting that the colour of virus-infected plants is more attractive to *B. tabaci* at least at short distances during the first steps of the host plant selection process.

It is well known that plant viruses frequently induce yellowing symptoms, and such was the case of tomato plants infected with ToSRV, which showed severe symptoms including yellowing, leaf rolling, interveinal chlorosis, and mottling. Tomato plants infected with ToCV showed milder symptoms, but yellowing and chlorosis were noticeable on the older leaves (Figure S1). Our results are consistent with those reported by Isaacs et al. [36] who found that *B. tabaci* orients its flight to green visual stimuli (light reflected at 550 ±10 nm) during foraging flight. Moreover, the preference of whiteflies for yellowing leaves such as symptomatic leaves of ToSRV- or ToCV-infected plants agrees with the early work by Mound [20] that found much more attraction of *B. tabaci* to deep yellow colour than to green or any other colour. The ventral half of the eye of *B. tabaci* is most sensitive to long—green-yellow—wavelengths (500–580 nm), which are in the region of earth’s reflecting energy. Conversely, the upper part is sensitive to short wavelengths (UV range: 340–380 nm), which belongs to the region of greatest visible sky energy [37]. Therefore, *B. tabaci* appears to locate its host plants using visual stimuli according to studies conducted so far that suggest vision as being the most important, if not the only cue used by *B. tabaci* in host-plant location from a distance [20,38].

Moreover, a number of studies were unable to find any role of olfactory cues in host plant finding ability of *B. tabaci*. Butler [39] for example, found that *Aleyrodes proletella* (L.) (Hemiptera: Aleyrodidae) was able to detect the smell from crushed cabbage leaves, while no response of *B. tabaci* was observed to several types of olfactory stimuli (crushed leaves or insect repellents) [20]. Such differences in the sensitivity to odours between the two species probably reflect differences in their host range. While *A. proletella* feeds exclusively on crucifers, *B. tabaci* is extremely polyphagous, explaining why olfactory response may provide more benefit to the former rather than to the latter whitefly species. Later work by Mellor and Anderson [40] showed that both *A. proletella* and *B. tabaci* have olfactory receptors (sensilla) unique to the flagellum of the antennae that possess external features (pits and longitudinal grooves), which are a prerequisite for an olfactory function.

In fact, Bleeker et al. [41] were the first to prove that *B. tabaci* responded to volatiles emitted by cultivated and wild tomato genotypes. Their work showed that terpene volatiles from wild tomatoes act as repellents to *B. tabaci*. More precisely, *B. tabaci* antennae responded to certain sesquiterpenes (zingiberene and cucumene), which also elicited avoidance behaviour. Li et al. found that limonene also had a repellent effect, while (E) 2-hexenal and 3-hexen-1-ol exhibited a prominent attraction to *B. tabaci* [42]. Therefore, these recent studies point out that *B. tabaci* apparently uses, besides visual, specific plant volatile cues for the initial stages of host selection.

Nevertheless, according to our results, vision seems to be more important than olfaction when *B. tabaci* needs to discriminate between virus-infected and mock-inoculated plants. Results of our dual-choice leaf experiments in the presence and absence of light prove that visual cues seem to be more important in the initial steps of the host plant selection process. Whiteflies did not respond to tomato leaves in the absence of light but clearly concentrated on tomato leaves when the light was turned on, as expected for an insect that has a diurnal flight behaviour [43]. However, when *B. tabaci* was exposed to plant odours carried by airflow it responded to olfactory stimuli (Figure 1), but such response was not too strong. In fact, the response of *B. tabaci* to volatiles in our study was weak. About 30% of individuals had no response at all, only 60% reacted to the tomato mock-inoculated plant, while 40% reacted to the blank (empty container).

Terpenes seem to be important in plant defence against whitefly attack and virus infection may affect their synthesis. Interestingly, infection of plants with the begomovirus *Tomato yellow leaf curl China virus* (TYLCCNV) suppressed terpene synthesis, making infected plants more susceptible to MEAM1 *B. tabaci* [44]. Similar results were observed on tomato plants infected with *Tomato yellow leaf curl virus* (TYLCV), that had a significant reduction in the concentration of the volatiles beta-myrcene, thymene, beta-phellandrene, caryophyllene, 4-carene, and alfa-humulene [45]. Our results also showed a significant reduction in the concentration of certain terpenes after infection of tomato plants with the begomovirus ToSRV, and some of them (e.g., terpinene, and α-phellandrene) are known to repel non-viruliferous *B. tabaci* [41]. However, such a reduction in the concentration of terpenes was not associated to an increased attraction of *B. tabaci* to ToSRV-infected plants (Figure 1). Instead, olfactometer assays revealed that viruliferous *B. tabaci* preferred volatiles emitted by mock-inoculated rather than those produced by ToSRV-infected plants. The observed vector behaviour likely favours ToSRV dispersal, as *B. tabaci* adults switch their olfactory preference after they acquire the virus and become attracted to healthy plants with higher concentrations of specific terpenes. Thus, our work suggests that begomoviruses can manipulate whitefly olfactory response by switching their preference to non-infected plants once they become viruliferous, a behaviour that is known to increase the rate of spread of the virus [46]. Nevertheless, odours of ToSRV-infected plants apparently do not influence the likelihood of whiteflies in acquiring viral particles.

Unlike ToSRV, non-viruliferous whiteflies preferentially oriented towards mock-inoculated plants over ToCV-infected plants. This behaviour can hamper the secondary spread of the virus because non-viruliferous insects that reach a field with ToCV-infected plants will have a preference for non-infected plants, a behaviour that would reduce the probability of virus acquisition by whiteflies. Furthermore, the behaviour of ToCV-viruliferous whiteflies will not favour virus transmission and dispersal because they could not discriminate between non-infected and ToCV-infected plants as opposed to what was found for ToSRV-*B. tabaci* interactions.

There are key differences in the transmission mechanism of the persistently-transmitted begomoviruses and the semi-persistently-transmitted criniviruses by whiteflies. Begomiviruses are circulative viruses that may have evolved together with its whitefly vector, *B. tabaci*, as they circulate and remain in the vector’s body for most of the insect lifespan. Conversely, criniviruses are retained on the cuticle for some hours resulting in a much less intimate interaction with its vector, because the virus never crosses any gut barrier. Furthermore, criniviruses are transmitted by other whitefly species besides *B. tabaci*. Thus, a direct effect of ToCV on its vector conducive to enhanced transmission would be less likely to occur than in the case of ToSRV. It seems that true manipulation requires tight virus-host-vector co-evolution and would thus be effective exclusively in very specific plant-virus vector species associations [3]. However, it should not be excluded that the differences observed in the preference for virus-infected vs. mock-inoculated plants between viruliferous and non-viruliferous whiteflies could also be a plant-mediated effect. Differences in the composition of infected and non-infected plants may have altered the preference of viruliferous and non-viruliferous whiteflies because whiteflies were subjected for 72 h to different feeding sources (virus infected vs. non-infected tomatoes) before running the experiments.

Virus-vector interactions can be beneficial to both the virus and the vector following a mutualistic type of relationship, but negative effects have also been reported in the case of *B. tabaci*. For example, the *B. tabaci* MED species benefits from its virus-vector interactions when feeding on plants infected with TYLCV [47], probably because the virus infection alters the nutritional quality of the host plant and suppresses the synthesis of herbivore-induced defensive enzymes [48]. However, the same virus, TYLCV, showed negative effects on its whitefly vector, *B. tabaci* MEAM1 species, such as a reduced fecundity and lifespan [49]. Despite the fact that TYLCV modifies the behaviour of its vector by making infected plants more attractive to non-viruliferous MED species of *B. tabaci*, the same virus has an opposite effect on the MEAM1 species that prefers healthy tomato plants over TYLCV-infected plants, a behaviour that probably has a negative impact on virus spread in the field [45]. Similarly, our studies show that viruses that differ in their mode of transmission, such as begomoviruses and criniviruses, may alter the behaviour of its whitefly vector in a completely different way, either by enhancing (ToSRV) or reducing (ToCV) virus spread. Our work also showed that both viruses alter tomato plant volatiles in opposite ways: by suppressing (ToSRV) or raising (ToCV) the amounts of specific terpenes.

It is known that insects locate, select, and accept their host plants by integration of a series of visual, chemical, tactile, and gustatory stimuli. Our work demonstrated that *B. tabaci* is first attracted to virus infected tomato plants (either by ToSRV or ToCV) during landing, probably using visual cues. However, it is likely that they switch their preference and become more attracted to mock-inoculated plants after landing according to the results of our olfactory tests (Figure 1). Our findings using Y-tube choice tests are consistent with the results obtained from a series of dual-choice settlement preference greenhouse experiments, where *B. tabaci* adults were released inside large cages containing virus-infected and mock-inoculated tomato plants. We found that viruliferous whiteflies preferred to settle on mock-inoculated over ToSRV-infected plants while non-viruliferous *B. tabaci* preferred mock-inoculated over ToCV-infected plants (Maluta, N. et al., unpublished results). Here, we evaluated the role of visual and olfactory cues on host selection by whiteflies; however, besides olfactory cues, gustatory traits can also play a role on whitefly preference between virus-infected and healthy plants. Studies on the feeding behaviour of *B. tabaci* on virus-infected and mock-inoculated tomato plants could help to find out if any gustatory cues are responsible for the rejection of virus-infected plants by *B. tabaci*.

## 5. Conclusions

The experiments conducted with the circulative persistent begomovirus, ToSRV, showed that the virus has co-evolved together with its vector, *B. tabaci*, to optimise its own spread. Viruliferous whiteflies showed a clear preference for non-infected plants, a behaviour that will speed up the intrinsic rate of the disease progress curve. However, this type of manipulation of vector behaviour by a plant virus was not observed for the case of the semi persistent crinivirus, ToCV, which is not specifically transmitted by *B. tabaci* and has a much less intimate virus-vector relationship.

## Figures and Tables

**Table 1:** Mean ± SE number of whiteflies* landing on (**a**) *Tomato chlorosis virus* (ToSRV)-infected and (**b**) *Tomato severe rugose virus* (ToCV)-infected tomato leaves, mock-inoculated tomato leaves, and brown background (blank) 6 h after release from the launch platform. Plexiglass sheets containing a coating of mineral oil were placed on top of the leaves to provide a uniform landing surface (65 × 65 cm) and avoid the confounding effects of leaf olfactory cues. Each assay was repeated four times.

(**a**)		
**Treatment**	**Non-Viruliferous (*n* = 48)**	**Viruliferous (*n* = 48)**
ToSRV-infected	8.85 ± 1.34 ^a^	8.98 ± 1.29 ^a^
Mock-inoculated	4.17 ± 0.76 ^b^	3.29 ± 0.44 ^b^
Blank	1.71 ± 0.40 ^c^	1.67 ± 0.24 ^c^
df	2141	2141
F	40.087	37.910
*P*	*<0.0001*	*<0.0001*
(**b**)		
ToCV-infected	8.35 ± 0.97 ^a^	7.48 ± 0.74 ^a^
Mock-inoculated	5.85 ± 0.68 ^b^	5.65 ± 0.86 ^b^
Blank	2.15 ± 0.32 ^c^	4.81 ± 0.88 ^b^
df	2141	2141
F	27.237	5.791
*P*	*<0.0001*	*0.0038*

* Mean values represent the number of whiteflies landing per target (11 × 11 cm). Means followed by the same letter within columns indicate no significant (*p* > 0.05) differences between treatments according to an ANOVA followed by a Fisher Protected LSD test. Data was transformed by ln(x+1) before analysis. Infection status of viruliferous whiteflies was verified by PCR as described in Section 2.1.

**Table 2:** *Bemisia tabaci* adult choice * (mean ± SE) in dual-choice arena with no active airflow under light and dark conditions.

Treatment (*n* = 20)	Light	Dark
Blank (no leaf)	0.75 ± 0.25	0.40 ± 0.13
Mock-inoculated	5.55 ± 0.76	0.55 ± 0.23
χ^2^	82.687	0.475
*P*	*<0.001*	*0.566*
ToCV-infected	4.00 ± 0.92	0.15 ± 0.11
Mock-inoculated	2.60 ± 0.61	0.05 ± 0.05
χ^2^	5.981	1.046
*P*	*0.195*	*0.369*
ToSRV-infected	3.05 ± 0.52	0.55 ± 0.22
Mock-inoculated	0.80 ± 0.27	0.30 ± 0.10
χ^2^	28.048	1.492
*P*	*<0.001*	*0.279*

* Mean values represent the average number of times that each whitefly entered the selected test surface covered by the nylon screen below the leaf surface over a 10 min period. A total of 20 individuals were released inside a Petri dish for each test.

**Table 3:** Volatile emission of mock-inoculated and virus-infected plants (mean ± SE ng·g^−1^ leaf tissue·h^−1^) * (**a**) mock-inoculated and ToSRV-infected plants and from (**b**) mock-inoculated and ToCV-infected plants.

(**a**)			
**Retention Time (min)**	**Compound**	**Mock**	**ToSRV**
8.65	α-Pinene	5.0 ± 0.8 ^a^	1.2 ± 0.2 ^b^
10.41	4-Carene	34.6 ± 5.1 ^a^	10.4 ± 2.8 ^b^
10.51	α-Phellandrene	5.1 ± 0.9 ^a^	1.0 ± 0.3 ^b^
10.84	Terpinene	1.2 ± 0.3 ^a^	0.3 ± 0.1 ^b^
11.17	β-Phellandrene	86.9 ± 14.7 ^a^	27.3 ± 6.6 ^b^
19.63	α-Copaene	0.9 ± 0.4 ^a^	1.0 ± 0.7 ^a^
23.35	Unidentified terpene 1	2.3 ± 0.7 ^a^	2.7 ± 0.4 ^a^
23.71	Unidentified terpene 2	46.0 ± 10.2 ^a^	61.3 ± 9.8 ^a^
(**b**)			
**RetentionTime (min)**	**Compound**	**Mock**	**ToCV**
8.62	α-Pinene	13.7 ± 2.0 ^a^	5.8 ± 1.2 ^a^
10.36	4-Carene	45.6± 8.4 ^a^	406.6 ± 226.4 ^a^
10.46	α-Phellandrene	8.7 ± 0.8 ^a^	63.7 ± 40.1 ^a^
11.12	β-Phellandrene	144.2 ± 24.7 ^a^	1091.2 ± 549.2 ^a^
14.13	Unidentified terpene 3	7.4 ± 1.2 ^a^	14.8 ± 5.8 ^a^
18.73	Unidentified terpene 4	2.3 ± 0.2 ^a^	18.4 ± 3.7 ^b^
19.63	α-Copaene	1.4 ± 0.3 ^a^	3.3 ± 1.1 ^a^
20.58	β-Caryophyllene	7.3 ± 0.2 ^a^	35.0 ± 4.1 ^b^
21.29	α-Humulene	3.1 ± 0.2 ^a^	11.0 ± 1.3 ^b^
23.69	Unidentified terpene 5	2.8 ± 1.4 ^a^	9.8 ± 0.6 ^b^

* Means followed by the same letter indicate no significant (*p* < 0.05) differences between treatments according to Student’s *t-*test or Welch’s test.

## Supplementary Materials

The following Figures are available online at www.mdpi.com/1999-4916/8/8/225/s1, Figure S1: Landing platform showing the 4 × (3 × 3) latin square design with the different targets of blank (brown), virus-infected and mock-inoculated leaves; Figure S2: (**a**) Flight platform used in the free-choice landing experiments; (**b**) Insect-proof cage with flight platform used for landing experiments; Figure S3: Dual-choice assay set-up to assess whitefly preference for virus-infected or mock-inoculated tomato leaves. Picture represents initial tests using a mock-inoculated leaf versus blank (no leaf) under light (**a**) and dark (**b**) conditions; Figure S4: Experimental set-up to assess whitefly response to volatiles emitted by virus-infected and mock-inoculated tomato plants; Figure S5: ARS^®^ volatile collection system connected to glass chambers used to extract volatiles from virus-infected and mock-inoculated plants (3 plants of each type).
