# Supplementary material for: Tomato Infection by Whitefly-Transmitted Circulative and Non-Circulative Viruses Induce Contrasting Changes in Plant Volatiles and Vector Behaviour
Source: Viruses. 2016 Aug 11;8(8):225. doi: 10.3390/v8080225 (PMC4997587; doi:10.3390/v8080225)
Supplement: Supplementary file 1 [file viruses-08-00225-s001.pdf]

Supplementary Materials: Tomato Infection by Whitefly-Transmitted Circulative and Non-Circulative Viruses Induce Contrasting Changes in Plant Volatiles and Vector Behaviour

Alberto Fereres, Maria Fernanda G. V. Peñaflor, Carla F. Favaro, Kamila E. X. Azevedo, Carol H. Landi, Nathalie K. P. Maluta, José Mauricio S. Bento and Joao R. S. Lopes

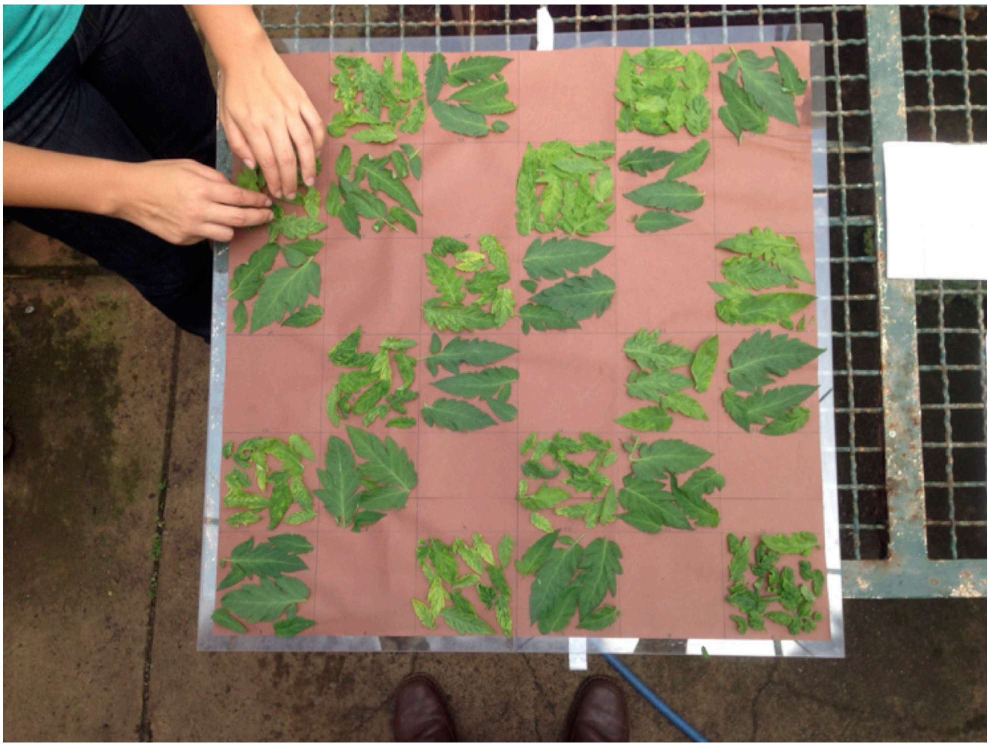

Figure S1. Landing platform showing the 4 × (3 × 3) latin square design with the different targets of blank (brown), virus-infected and mock-inoculated leaves.

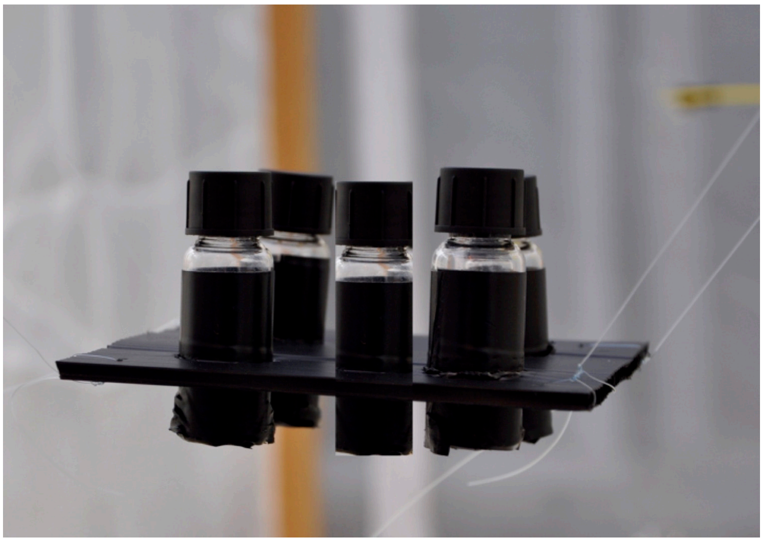

(**a**)

Figure S2. *Cont.*

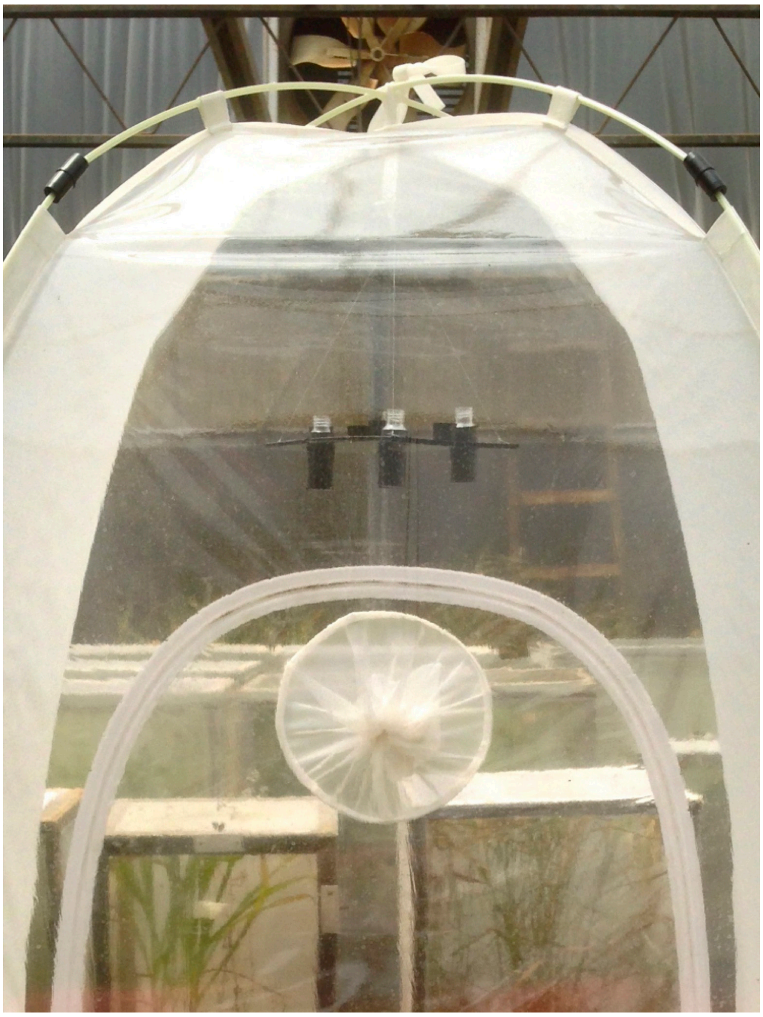

(**b**)

Figure S2. (**a**)Flight platform used in the free-choice landing rate experiments; (**b**) Insect-proof cage with flight platform used for landing experiments.

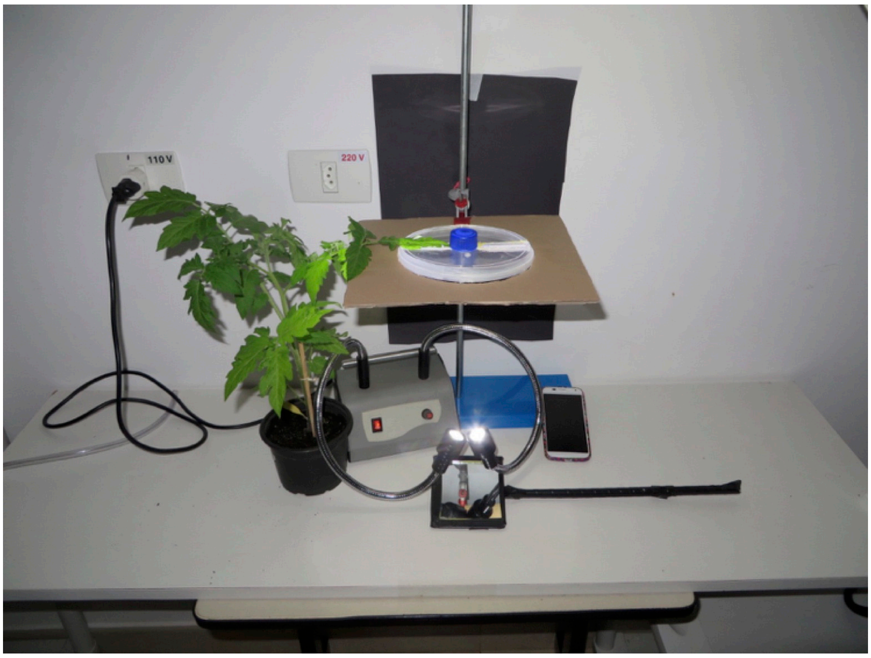

(**a**)

Figure S3. *Cont.*

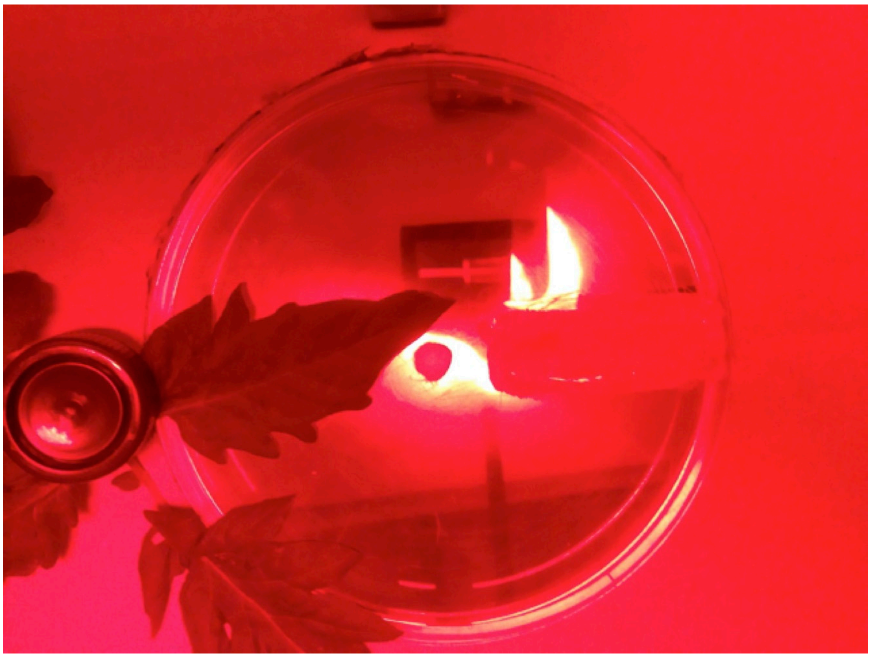

(**b**)

Figure S3. Dual-choice assay set-up to assess whitefly preference for virus-infected or mock-inoculated tomato leaves. Picture represents initial tests using a mock-inoculated leaf versus blank (no leaf) under light (**a**) and dark (**b**) conditions.

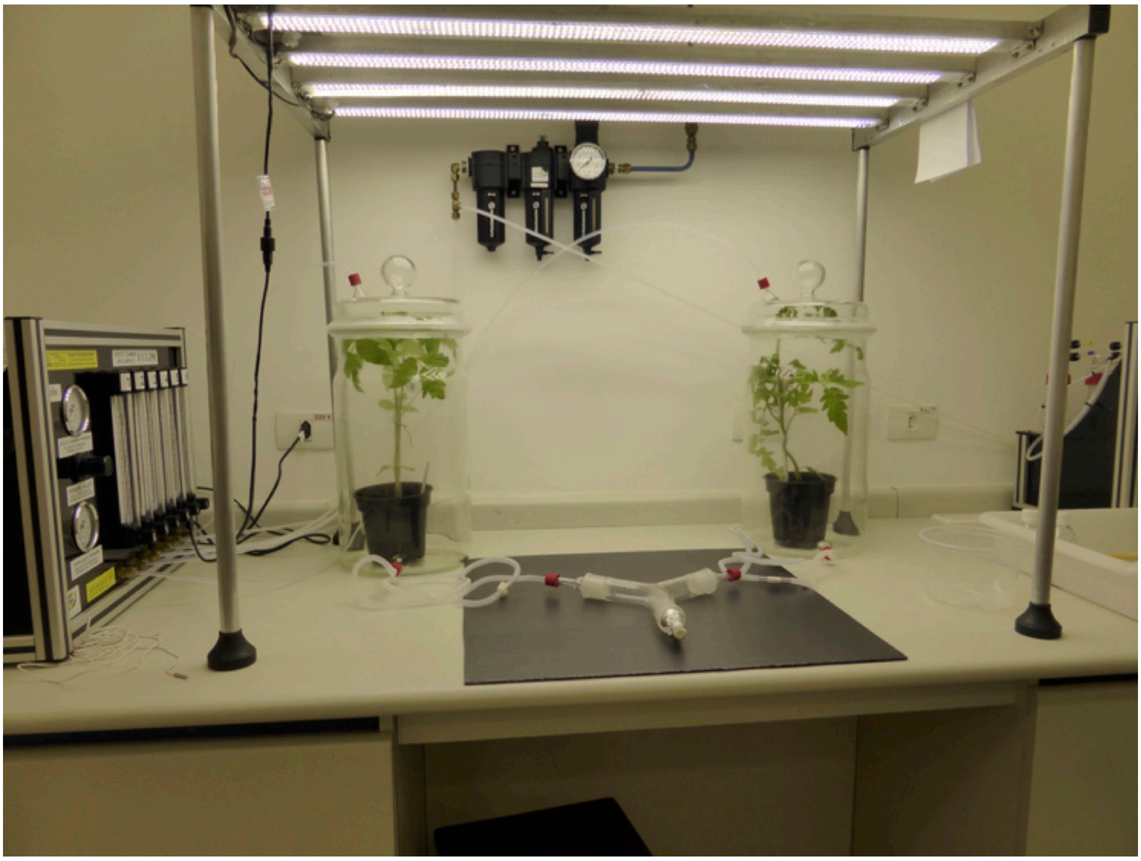

Figure S4. Experimental set-up to assess whitefly response to volatiles emitted by virus-infected and mock-inoculated tomato plants.

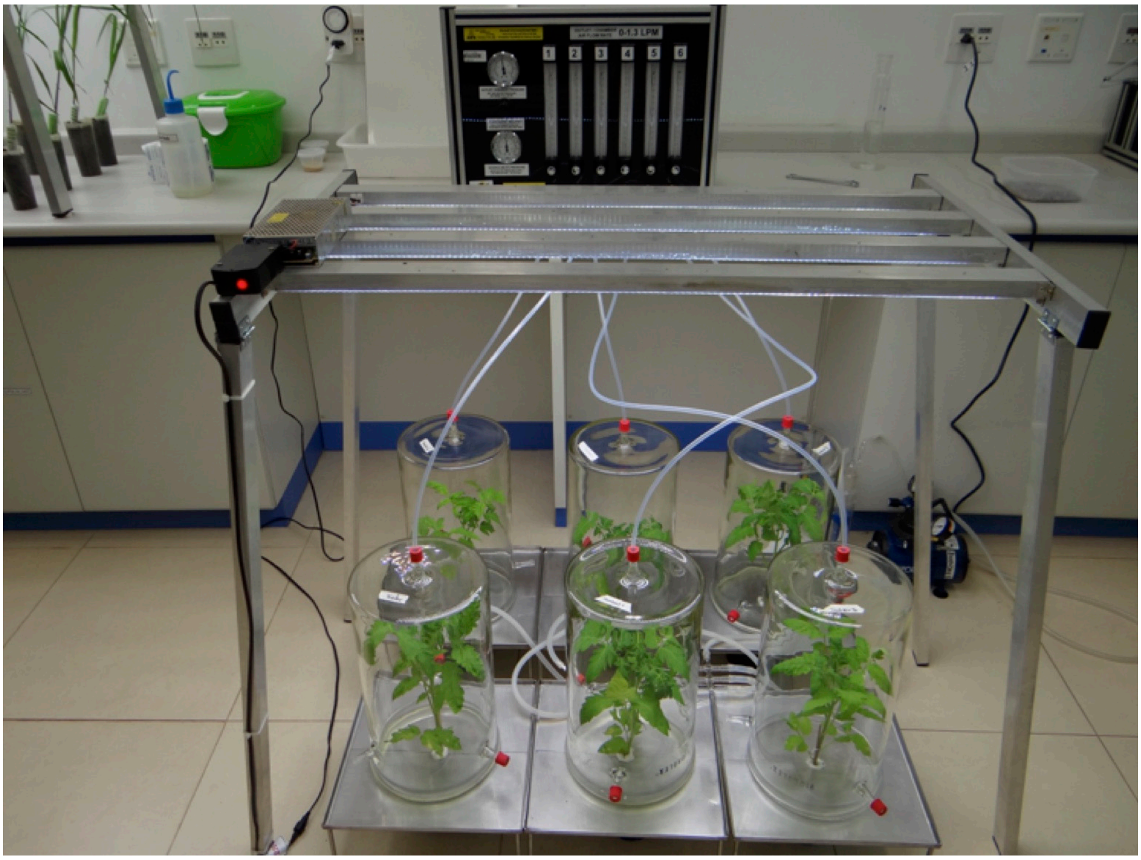

Figure S5. ARS® volatile collection system connected to glass chambers used to extract volatiles from virus-infected and mock-inoculated plants (3 plants of each type).

© 2016 by the author; licensee MDPI, Basel, Switzerland. This article is an open access article distributed under the terms and conditions of the Creative Commons Attribution (CC-BY) license (http://creativecommons.org/licenses/by/4.0/).
